# Optimizing the Dryland Sheet Erosion equation in South China

**DOI:** 10.1038/s41598-022-09258-x

**Published:** 2022-04-15

**Authors:** Dongdong Wang, Zaijian Yuan, Dingqiang Li, Yong Chen, Zhenyue Xie, Yanfei Lai

**Affiliations:** 1 National-Regional Joint Engineering Research Center for Soil Pollution Control and Remediation in South China, Guangdong Key Laboratory of Integrated Agro-environmental Pollution Control and Management, Guangdong Engineering Research Center for Non-point Source Pollution Control, Institute of Eco-environmental and Soil Sciences, Guangdong Academy of Sciences, Guangzhou, 510650 People’s Republic of China; 2 College of Ecology, Resources and Environment, Dezhou University, Dezhou, 253023 Shandong China; 3 State Key Laboratory of Soil Erosion and Dryland Farming On the Loess Plateau, Institute of Soil and Water Conservation, Northwest A&F University, Yangling, Shaanxi 712100 China; 4 International Institute of Soil and Water Conservation, Meizhou, 514000 People’s Republic of China; 5 Wuhua Soil and Water Conservation Expanding Station of Guangdong Province, Meizhou, 514471 China

**Keywords:** Ecology, Plant sciences, Ecology, Hydrology

## Abstract

Optimisation of models applied in sheet erosion equations could facilitate effective management of sheet erosion in the field, and sustainable agricultural production. To optimise the characterisation of sheet erosion on slope farmland in South China, the present study conducted field simulation rainfall experiments with vegetated and fallow soils. According to the results, sheet erosion rate first increased with an increase in rainfall duration and then stabilised. Exclusive *P. vulgaris* planting and *P. vulgaris* in combination with earthworms could reduce sheet erosion by 10–60%, and the combined method could better control sheet erosion. There were significant differences in erosion rate between mild and steep slopes, and light and heavy rain conditions. The influence of rain intensity on sheet erosion was greater than that of slope. Soil organic matter (SOM), rain intensity, and slope can be used to optimise sheet erosion equations of exposed slopes, and SOM and hydraulic parameters can be used to optimise sheet erosion equations in vegetated slopes. The results of the present study could facilitate the reduction of the time and space variability errors in the establishment of sheet erosion models for vegetated slopes.

## Introduction

Sloping farmlands in China, which are mainly distributed in hilly areas, occupy a large proportion of the agricultural land in the country. The hilly area in South China experiences frequent geological disasters such as debris flows, which are primarily caused by the accumulation and evolution of sheet erosion over large spatial scales^1,2^. During the fallow period in cultivated land, erosion is severe in bare slopes, and numerous studies have explored the effects of bare slopes on erosion^3^. However, slope erosion rate changes considerably following crop cultivation on agricultural land after a fallow period, and few studies have explored^4,5^. Research on the optimization and characterization of dry land in South China is necessary and important.

Both external (slope and rain intensity) and internal factors (hydraulic parameters) influence sheet erosion^6,7^. Generally, sheet erosion rate increases with an increase in slope or rain intensity, which can be described based on a linear or power function^6,8^ Hydraulic parameters are indicators of the driving force of soil erosion, and sheet erosion rate increases with an increase in hydraulic parameters. Researchers often use the three hydraulic parameters of shear stress, stream power, and unit stream power to describe sheet erosion. Under bare slope conditions, sheet erosion can be described based on a power function of stream power or a power function of shear stress^8^. Under grassland conditions, sheet erosion can be described based on a power function of stream power^9,10^. However, controversy persists on the degree of influence of slope or rain intensity on sheet erosion, and temporal and spatial changes make the description and prediction of sheet erosion in different treatments challenging^11,12^.

Planting crops improves soil roughness, runoff resistance coefficient, and soil infiltration rate by improving soil properties, in addition to reducing soil erodibility and runoff on planted slopes^13^.Compared to in the fallow period, soil erosion rate in cultivated and planted land is significantly different, which is also related material and energy cycling in the agricultural land^14^. The integration of soil properties to the sheet erosion equation could optimise the characterisation of sheet erosion.

In the present study, three farmland treatments were set up, including no crops (simulated fallow farmland period), single planting of *Prunella vulgaris* (simulated cropping period in farmland), and *P. vulgaris* combined with earthworms (simulated farmland planting period). The objectives of the present study were to (1) analyse sheet erosion rate under rainfall; (2) analyse the responses of sheet erosion rate to rain intensity or slope, and establish empirical equations for sheet erosion rate based on slope rain intensity or slope in different treatments; (3) analyse the responses of sheet erosion rate to hydraulic parameters and establish the empirical equations for sheet erosion rate in different treatment slopes using hydraulic parameters; and (4) optimise sheet erosion rate equations by integrating soil indicators. The results of the present study could facilitate sheet erosion prediction and evaluation.

## Materials and methods

### Study area

The Guangdong Wuhua Soil and Water Conservation Science and Technology Demonstration Park is located in Wuhua County, in the eastern part of Guangdong Province, between 23°23–24°12 N latitude and 115°18–116°02 E longitude. The geology is complex, with hills accounting for 41.3% of the total area. Wuhua County has a humid monsoon climate in the middle and low latitudes of the southern subtropical zone, with an average annual temperature of 21.2 °C and average annual rainfall of 1519.7 mm.

### Experimental plot layouts

**Figure 1:**
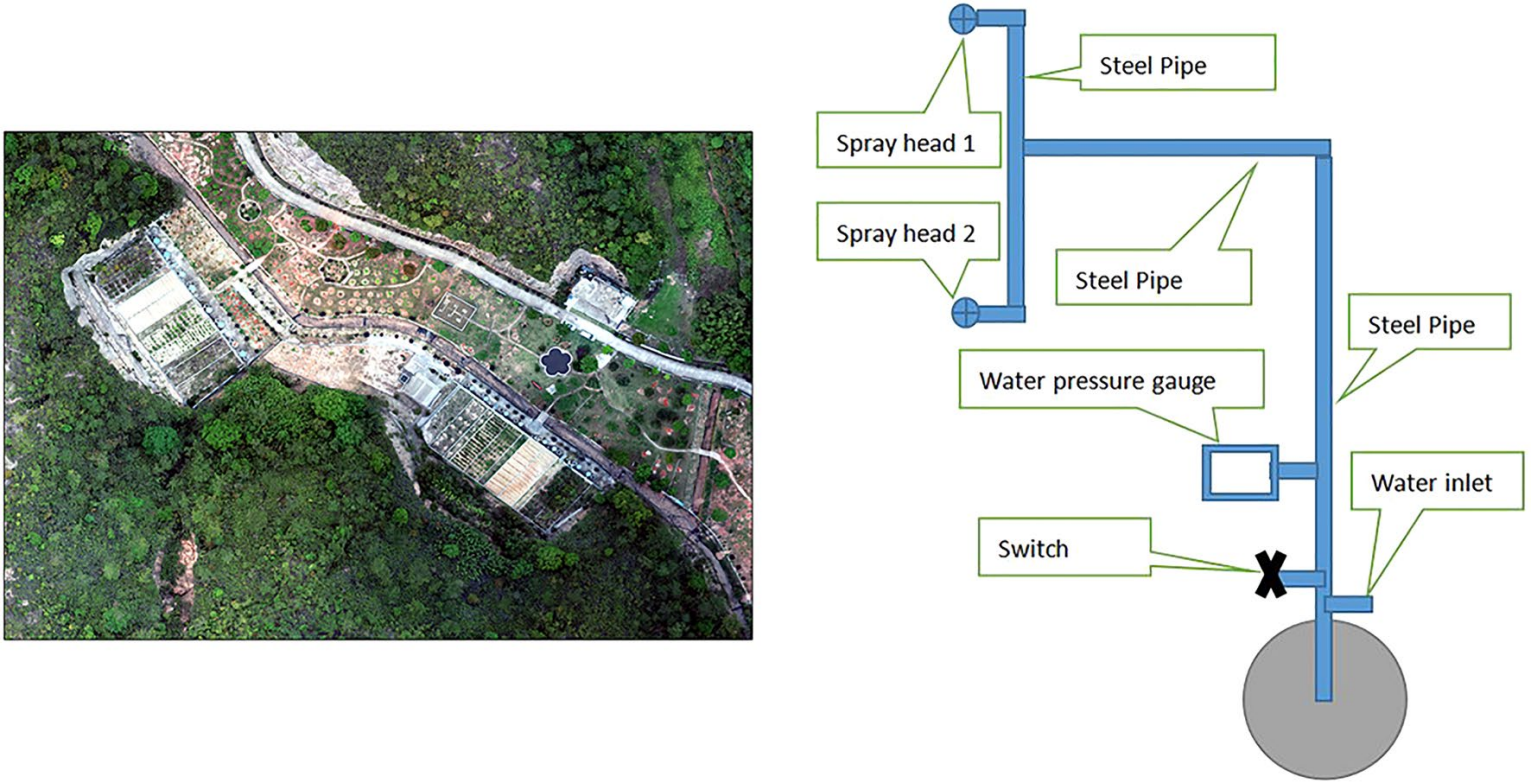
The experiment site and the rainfall systems.

Field tests and indoor tests were conducted separately at the Guangdong Wuhua Soil and Water Conservation Science and Technology Demonstration Park (Fig. 1) and Guangdong Environmental Science and Technology Public Laboratory. The equipment used included portable rainfall systems (Fig. 1), laser rain spectrometers, and digital cameras. The plant materials included *P. vulgaris L*. (plant spacing of 15 cm × row spacing of 20 cm) and *Eisenia foetida* (5 g/piece, 400 g/m^2^).The length and width of the simulated rainfall test plot was 1.2 m × 1.0 m, and each plot was separated by concrete partitions to limit the free movement of earthworms that we maintained the required density of earthworms per unit area of the plot.. After ploughing and transplanting *P. vulgaris* on the sample plots and introducing earthworms, no farming was carried out. The slope was designed according to the local topography and the rain intensity was designed according to the rainfall in the area in the past 50 years. The test treatment and the number of sessions are listed in Table 1.

**Table 1:** Field simulation rainfall test design and times.

Treatment	O	B	A
Level	Carry out 9 fields each with earthworm treatment and bare plots at a slope of 10° at 0.7, 1.2, 1.6, 2.2, 2.7 mm/min or at a slope of 2°, 5°, 15°, 20° at a rain intensity of 1.6 mm/min		According to the rain intensity of 1.6, 2.2 and 2.7 mm/min at a slope of 10° or 1.6 mm/min rain at a slope of 15° and 20°, only Prunella vulgaris was planted for 4 plots
Repetition	1	1	1
Number	18	18	8
Total	44		

The significance level of the equation is 0.05. O is bare slope, B is Prunella vulgaris combined with earthworm planting slope, A is Single planting prunella vulgaris slope.

### Testing and data collection

**Figure 2:**
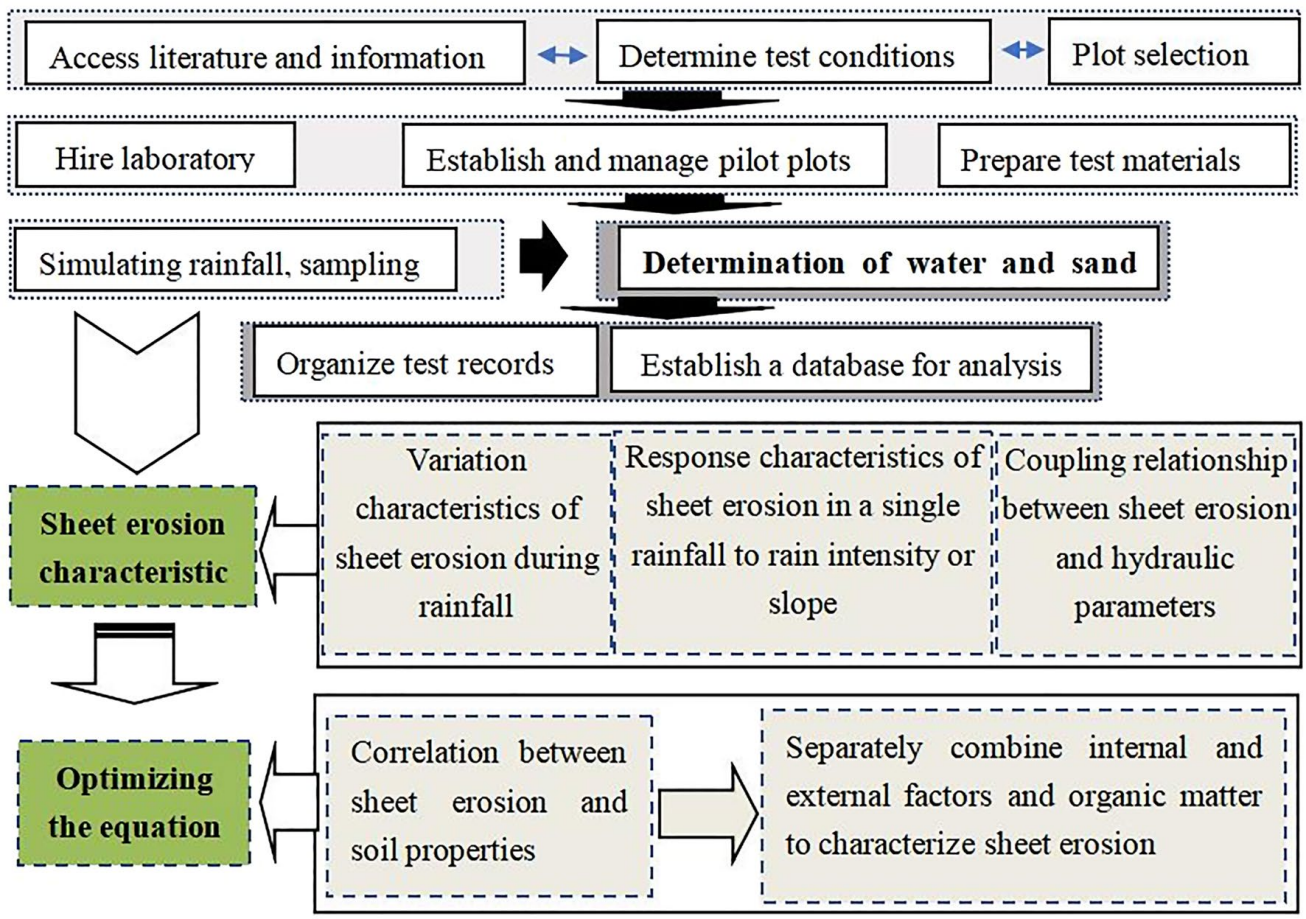
The schematic of the study.

After the planting of *P. vulgaris* was stabilised, rainfall simulation was carried out in the field. Rain intensity was adjusted according to the test plan, samples were obtained every 3 min, and the last sample was obtained at the end of the rainfall event, which lasted 40 min. Before sampling, the dye method was used to measure flow velocity in the velocity measurement area over a distance of 50 cm. Flow velocity was measured once on each side of the velocity measurement area, and the average value represented the flow velocity during the sampling period. When it rains, a thermometer is used to measure the temperature of the muddy water, and a laser rain spectrometer is used to measure the size and end speed of the raindrops. A small bucket was used to collect all the water and sand (the dried sand is sheet erosion and splash erosion), measure the volume of the muddy water with a graduated cylinder, and then clarify, skim off the water, dry, and weigh heavily. Figure 2 presents a schematic of the study.

The soils in sampling plots were latosolic red soil. The pH value was assessed using a pH meter, and organic matter, soil bulk density, soil particle density, and total water-soluble salts were measured according to the NY/T 1121.6–2006, NY/T 1121.4–2006, NY/T 1121.23–2010, and NY/T 1121.16–2006 Chinese agricultural industry standards, respectively. Conductivity was tested according to the HJ 802–2016 Chinese National Environmental Standard. Microbial carbon was tested using the chloroform fumigation-potassium sulphate extraction-potassium dichromate bulk density method, and particle composition was tested using the hydrometer method. The above indicators are shown in Table 2. Soil particle compositions of the sample plots are shown in Table 3.The four hydraulic parameters were calculated using the following formula:

1 $$\tau = \rho g{\text{h}}S$$

where τ is shear stress (Pa)^15^, ρ is the water density (kg m^−3^), g is the gravitational acceleration (m s^−2^), h is the flow depth (m), and *S* is the sine value of the slope gradient;

2 $$\omega = \tau V = \rho ghSV,$$

where ω is the stream power (W m^−2^;)^16^, V is the mean flow velocity (m s^−1^) and

3 $${\text{U}} = {\text{VS}}.$$

where U is unit stream power^17^;

**Table 2:** Soil property indicators of sample plots. S is Slope (^o^),BD is soil bulk density(g/cm^3^), OM is organic matter(g/kg), SD is soil particle density(g/cm^3^), CD is electrical conductivity(ds/m), WS is water-soluble salt content(g/kg), MC is microbial carbon(mg/kg), P_1_ is > 2 mm soil particle content(%), P_2_ is < 0.002 mm soil Particle content(%). O is bare slope, B is Prunella vulgaris combined with earthworm planting slope, A is Single planting prunella vulgaris slope.

Treatment	S	BD	pH	OM	SD	CD	WS	MC	P_1_	P_2_
O	2	1.26	4.80	16.30	2.59	0.053	0.2	82.2	36.19	24.44
	5	1.21	5.40	15.60	2.60	0.045	0.3	78.8	40.05	14.27
	10	1.18	4.56	11.60	2.64	0.150	0.8	72.2	41.47	12.27
	15	1.15	5.84	7.80	2.60	0.130	0.9	68.8	41.67	11.88
	20	1.12	6.47	6.90	2.61	0.080	0.5	66.8	46.88	13.29
A	10	1.38	5.40	15.60	2.60	0.045	0.3	225.0	31.25	23.51
	15	1.34	4.56	11.60	2.64	0.150	0.8	158.0	34.44	22.42
	20	1.32	5.84	7.80	2.6	0.130	0.9	131.0	36.19	24.44
B	2	1.47	5.35	23.70	2.56	0.059	0.9	232.0	30.35	24.42
	5	1.46	4.76	19.60	2.58	0.120	0.8	225.0	34.25	24.15
	10	1.41	4.80	16.30	2.59	0.053	0.2	158.0	33.44	21.42
	15	1.37	5.40	15.60	2.6	0.045	0.3	131.0	35.01	23.86
	20	1.34	4.56	11.60	2.64	0.150	0.8	90.2	35.52	24.39

**Table 3:** Representative values of soil mechanical composition testing. O is bare slope, B is Prunella vulgaris combined with earthworm planting slope, A is Single planting prunella vulgaris slope.

Treatment	Content of soil particle composition of each particle size (mm%)							
	> 2	2.0 ~ 1.0	1.0 ~ 0.5	0.5 ~ 0.2	0.2 ~ 0.05	0.05 ~ 0.02	0.02 ~ 0.002	< 0.002
O	40.05	9.87	21.09	16.24	15.82	2.01	20.70	14.27
A	36.19	8.93	17.21	12.42	14.17	4.04	18.79	24.44
B	30.35	11.04	18.35	10.90	15.51	2.02	17.76	24.42

The unit energy (E, measured in cm)^18^ was calculated as follows:

4 $$E = \alpha V^{2} (2g)^{ - 1} + h\cos \theta ,$$

where ɑ is the kinetic energy correction factor (ɑ= 1) and θ is the slope angle (°).

### Data processing

Photoshop (Adobe Inc., San Jose, CA, USA), IBM SPSS 19.0 (IBM Corp., Armonk, NY, USA), and MS Excel 2003 (Microsoft Corp., Redmond, WA, USA) were used to make graphs and tables. Three data analysis methods, including Analysis of Variance, regression analysis, and time series analysis were used to analyse the characteristics of sheet erosion during the rainfall process, using 616 datasets(average value) ; Analysis of Variance, regression analysis, Least Significant Difference (LSD) test, and interval estimation F test were used to analyse the response characteristics of the sheet erosion rate of a single rainfall event to internal and external factors, using 44 datasets(average value)^13,14^.

## Results

### Variation characteristics of sheet erosion during rainfall

**Figure 3:**
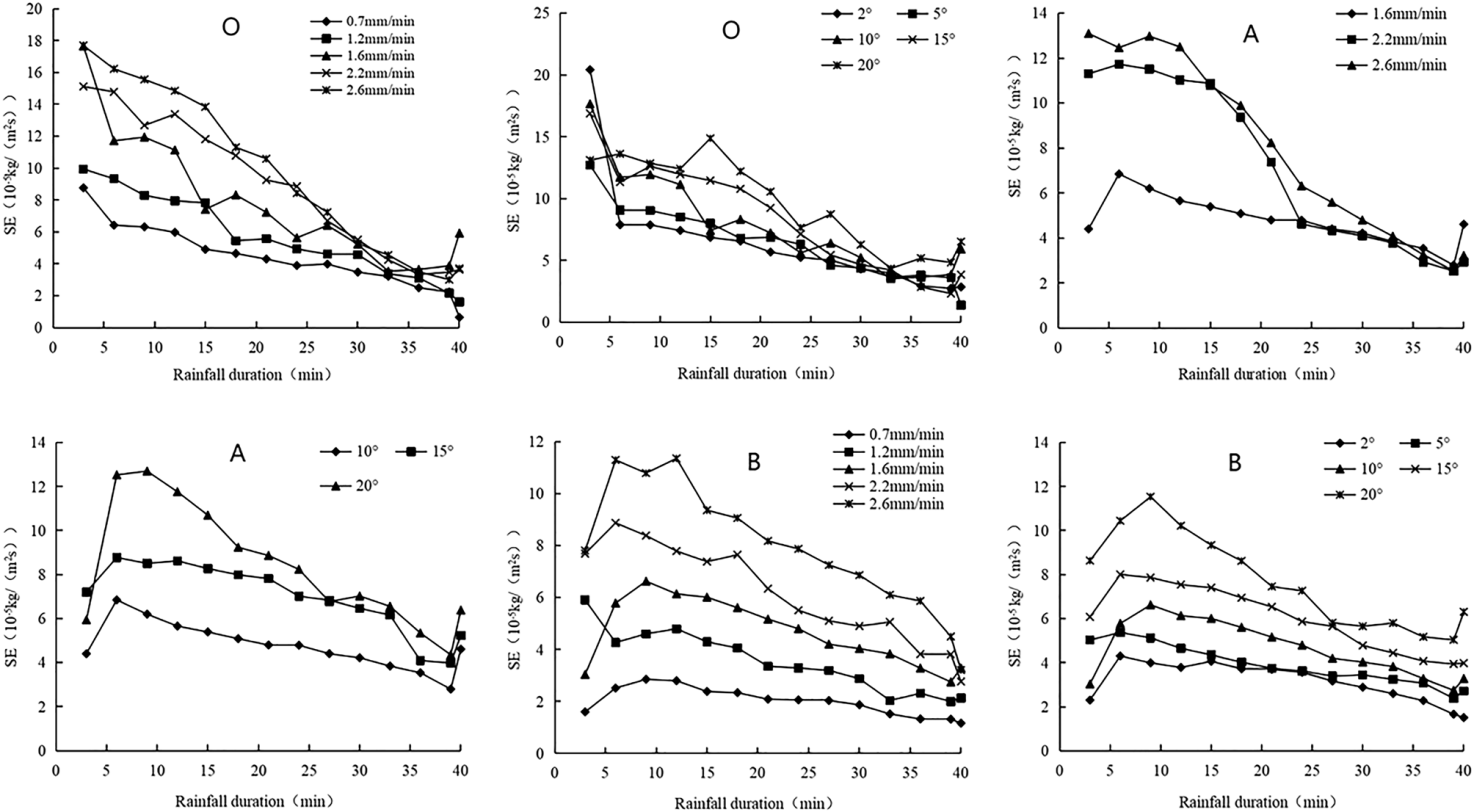
Variations of sheet erosion rate with rainfall duration in different treatments. O is bare slope, B is Prunella vulgaris combined with earthworm planting slope, A is Single planting prunella vulgaris slope.

Sheet erosion rate decreased as the rainfall time increased and then stabilized, under different rain intensities or slopes, with significant fluctuations in 0–6 min. The sheet erosion rates of the bare slope, *P. vulgaris* slope, and in the *P. vulgaris* combined with earthworm slope were stable at 30, 33 , and 37 min, respectively, at 2 × 10^–5^ ~ 6 × 10^–5^ kg/(m^2^ s), 1 × 10^–5^ ~ 5 × 10^–5^ kg/(m^2^ s), 0.5 × 10^–5^ ~ 6 × 10^–5^ kg/(m^2^ s), respectively (Fig. 3). Differences in sheet erosion rate among the three treatments based on rain intensity or slope decreased with an increase in rainfall time, and the differences between rain intensity treatments were greater than those between slope treatments. However, compared to that of the bare slopes, the erosion rate in the two planted slopes decreased under rainfall, and the index value decreased during the stable period. *P. vulgaris* in combination with earthworms had the most obvious effect. The soil erosion during the fallow period was severe compared to that under cropping, and the intensity and volatility of soil erosion were reduced significantly under cropping, and the ecological planting mode was better. Thirty minutes before rainfall, is the key to preventing and controlling soil erosion.

### Response characteristics of sheet erosion to rain intensity and slope

**Figure 4:**
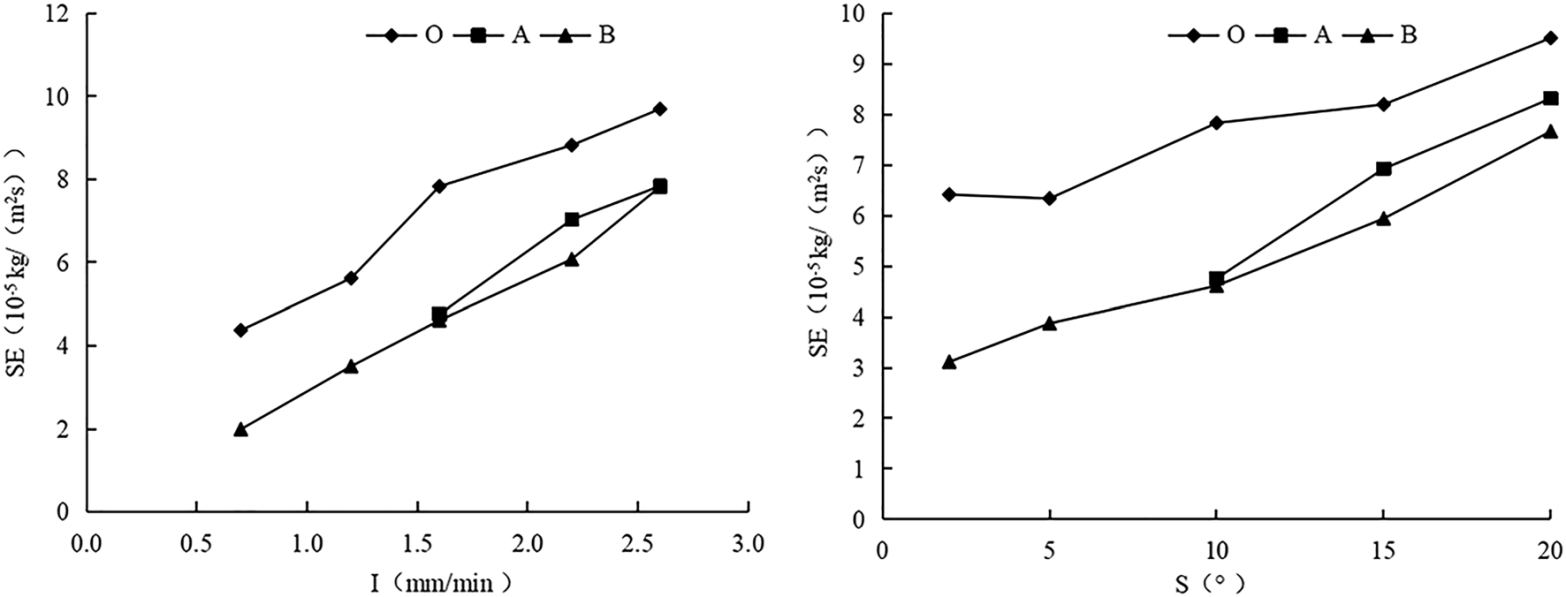
Variations of sheet erosion rate of three treatments with rain intensity or slope. O is bare slope, B is Prunella vulgaris combined with earthworm planting slope, A is Single planting prunella vulgaris slope.

Increasing slope from 2° to 20° or rain intensity from 0.7 to 2.6 mm/min increased sheet erosion rate gradually (Fig. 4 and Table 4) , which can be described based on a binary function equation, with a coefficient of determination (R^2^) > 0.9 (Table 5). The coefficients of rain intensity in the sheet erosion equations under different treatments were more than tenfold the coefficients of slope in the sheet equation (Table 5). In addition, there were significant differences in the sheet erosion rate between gentle (2° or 5°) and steep (15° or 20°) slope, light (0.7 mm/min or 1.2 mm/min) and heavy (2.2 mm/min or 2.6 mm/min) rain (Table 4). The regulating effect of the two planting methods on sheet erosion rate was 10–60% (Table 4). The effect of *P. vulgaris* in combination with earthworms in reducing sheet erosion was greater than that of exclusive *P. vulgaris* planting (Table 4). Overall, the influence of rain intensity on sheet erosion rate was greater than the influence of slope on sheet erosion rate, and the ecological planting method could better limit sheet erosion.

**Table 4:** Sheet erosion rates and their regulating effects of two planting methods.

S (°)/I (mm/min)		SE (kg/(m^2^ s))			Reduction (%)	
		O	B	A	B	A
10	0.6	4.37a	1.99a		54.51	
	1.2	5.62ab	3.51b		37.58	
	1.6	7.83bc	4.61b	4.76a	41.12	39.27
	2.2	8.83bc	6.08c	7.03ab	31.15	20.34
	2.6	9.70c	7.82d	7.84b	19.33	19.16
1.6	2	6.42a	3.11a		51.47	
	5	6.34a	3.87ab		38.86	
	10	7.83ab	4.61b	4.76a	41.12	39.27
	15	8.20ab	5.94c	6.93b	27.54	15.51
	20	9.51b	7.67d	8.32b	19.41	12.58

a, b, c, d are the difference comparison of LSD , and the significance level is 0.05. O is bare slope, B is Prunella vulgaris combined with earthworm planting slope,A is Single planting prunella vulgaris slope.

**Table 5:** The relationship between the sheet erosion rate and the slope or rain intensity**.**SE is sheet erosion rate, S is Slope (^o^),I is Rainfall intensity(mm/min).

Treatments	Equations
O	SE = 9.43 × 10^–6^ + 1.80 × 10^-6^S + 2.84 × 10^-5^I, R^2^ = 0.9472
B	SE = − 2.34 × 10^–5^ + 2.45 × 10^-6^S + 2.93 × 10^-5^I, R^2^ = 0.9800
A	SE = − 9.85 × 10^–5^ + 5.06 × 10^–5^ ln(S) + 6.39 × 10^–5^ ln(I), R^2^ = 0.9942

The significance level of the equation is 0.05. O is bare slope, B is Prunella vulgaris combined with earthworm planting slope,A is Single planting prunella vulgaris slope.

### Relationship between sheet erosion and hydraulic parameters

**Figure 5:**
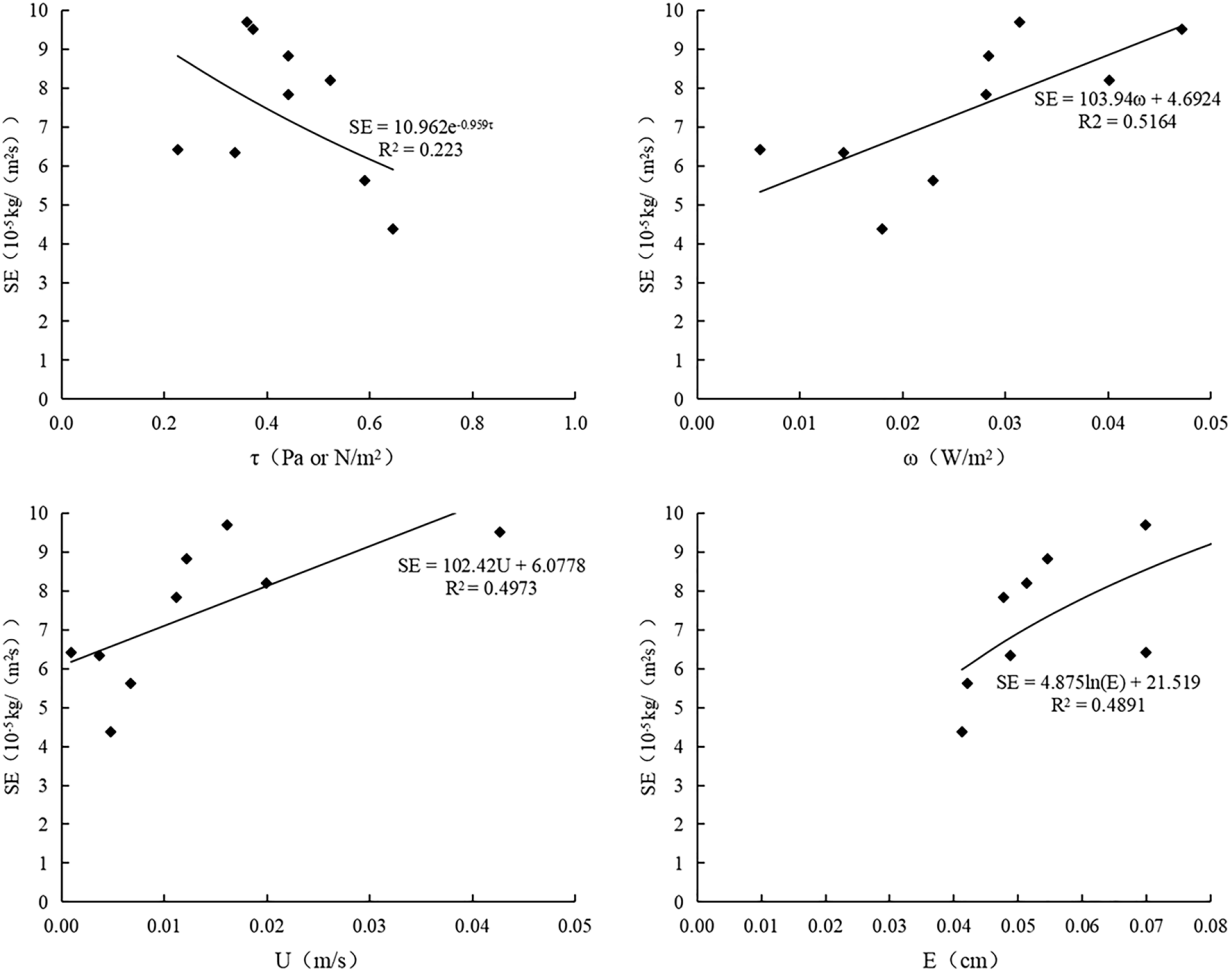
Variations of sheet erosion rate of exposed slope with hydraulic parameters. SE is sheet erosion rate, O is bare slope, B is Prunella vulgaris combined with earthworm planting slope, A is Single planting prunella vulgaris slope.

**Figure 6:**
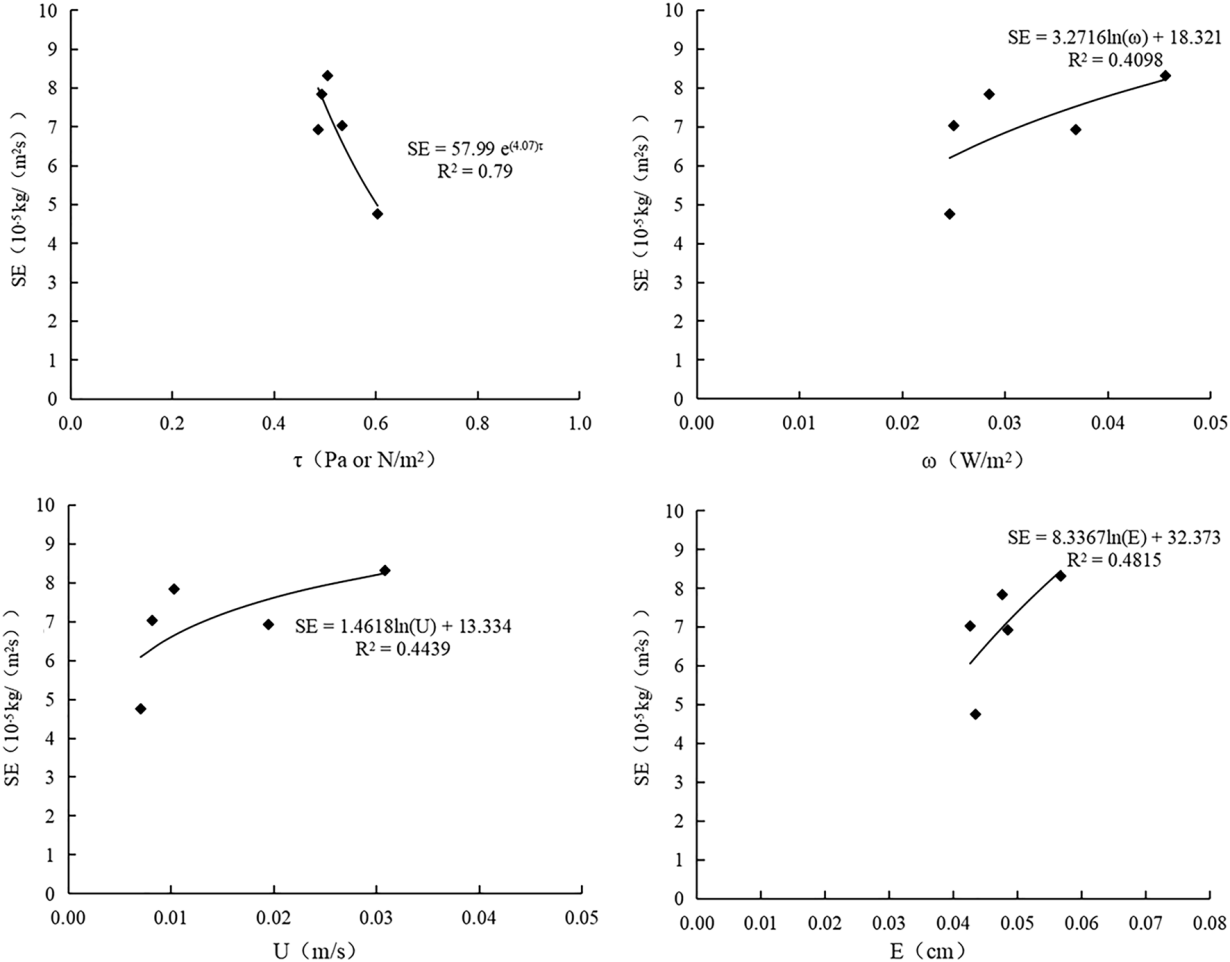
Variations of sheet erosion rate of Prunella vulgaris planting slope with hydraulic parameters. O is bare slope, B is Prunella vulgaris combined with earthworm planting slope, A is Single planting prunella vulgaris slope.

**Figure 7:**
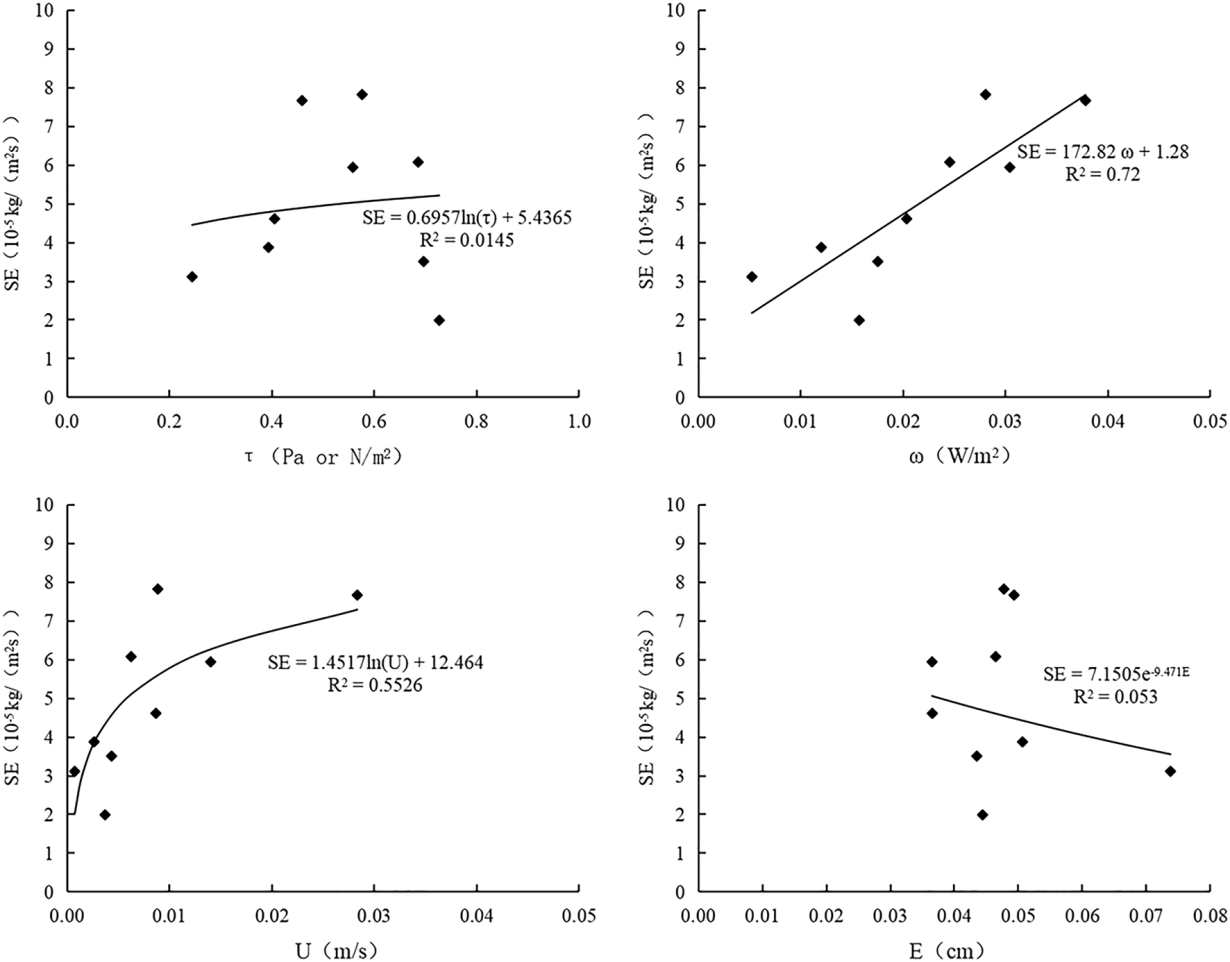
Variations of sheet erosion rate of prunella vulgaris combined with earthworm slope with hydraulic parameters. O is bare slope, B is Prunella vulgaris combined with earthworm planting slope, A is Single planting prunella vulgaris slope.

Sheet erosion rates in different slope treatments increased with an increase in hydraulic parameter. In bare slopes, exclusive *P. vulgaris* slopes, and *P. vulgaris* in combination with earthworm slopes, sheet erosion rates had a linear relationship with stream power, exponential relationship with shearing force, and linear relationship with stream power, with R^2^ values of 0.52, 0.79, and 0.72, respectively (Fig. 5, 6, 7). The fitting effect of sheet erosion for bare soil is general.

### Optimized characterization of sheet erosion

Previous studies have shown that soil organic matter (SOM) is a key indicator of the sensitivity of soil to erodibility^19,20^, and that SOM influences surface runoff by improving soil infiltration rate and surface roughness^21,22^. The comprehensive role of soil organic matter in erosion is an external factor (slope and rain intensity)and the hydraulic parameters cannot be reflected. Comprehensive consideration of soil properties would facilitate the optimisation of the characterisation of sheet erosion .

The present study analysed the correlation between slope erosion rate under different slope, rainfall, and soil property conditions. Organic matter had good correlation with erosion rates in slopes under different treatments, with an R^2^ of approximately 0.5 (Table 6). According to the results, organic matter characterises sheet erosion optimally among various soil indices.

**Table 6:** Analysis of Correlation between sheet erosion rate and soil properties of three treatments. The significance level of the equation in the table is 0.05.

Treatments		Index								
		Soil properties								
		BD	pH	OM	SD	CD	WS	MC	P_1_	P_2_
O	SE	− 0.36	0.22	− 0.43	0.23	0.45	0.47	− 0.38	0.24	− 0.25
B		− 0.61	− 0.22	− 0.57	0.61	0.28	− 0.04	− 0.59	0.62	− 0.50
A		− 0.51	0.25	− 0.54	− 0.02	0.38	0.47	− 0.50	0.51	0.33

BD is soil bulk density(g/cm^3^), OM is organic matter(g/kg), SD is soil particle density(g/cm^3^), CD is electrical conductivity(ds/m), WS is water-soluble salt content(g/kg), MC is microbial carbon(mg/kg), P_1_ is > 2 mm soil particle content(%), P_2_ is < 0.002 mm soil Particle content(%). O is bare slope, B is Prunella vulgaris combined with earthworm planting slope, A is Single planting prunella vulgaris slope.

When organic matter was integrated in a sheet erosion equation including rain intensity or slope, R^2^ of the sheet erosion equation associated with bare soil increased by 0.0235, and the change was significant. The R^2^ values of the exclusive *P. vulgaris* and *P. vulgaris* in combination with earthworm treatments increased by 0.0003 or 0.0042, respectively, showing minimal change (Table 7). When organic matter was added to the sheet erosion equation including hydraulic parameters, the R^2^ value of the sheet erosion equation associated with bare soil increased by 0.0014, which was a minor change. Conversely, R^2^ values of the exclusive *P. vulgaris* and *P. vulgaris* in combination with earthworm treatments increased by 0.0015 and 0.1637, respectively, which are considerable changes (Table 7). Consequently, the combination of organic matter combined with rain intensity or slope significantly optimised the bare soil sheet erosion equation, and organic matter in combination with hydraulic parameters optimised the cropped slope sheet erosion equation.

**Table 7:** The optimized sheet erosion equations for three slope treatments.O is bare slope, B is Prunella vulgaris combined with earthworm planting slope, A is Single planting prunella vulgaris slope.

Treatments	Equations	Difference
O	SE = 0.00018 × S^-0.021^ × I^-0.629^ × OM^-0.473^, R^2^ = 0.9707	0.0235
	SE = 4.27 × 10^–5^ + 0.001ω + 2.23 × 10^−7^OM, R^2^ = 0.5178	0.0014
B	SE = − 5.2 × 10^−5^ + 3.19 × 10^−6^S + 2.94 × 10^−5^I + 1.23 × 10^−6^OM, R^2^ = 0.9842	0.0042
	SE = − 0.00012 + 0.0034ω + 5.66 × 10^−6^OM, R^2^ = 0.8837	0.1637
A	SE = − 0.0001 + 5.65 × 10^−5^ ln(S) + 6.45 × 10^−5^ ln(I) + 5.93 × 10–6 ln(OM), R^2^ = 0.9945	0.0003
	SE = 0.00056e^−3.79τ−0.008OM^, R^2^ = 0.8052	0.0015

## Discussion

Soil in the red soil in South China is characterised by coarser particles (particle size > 2 mm) and fewer fine particles (particle size < 0.002 mm)(Table 3), and rainfall erodes the soil through two types of raindrops and runoff^23,24^. Initial raindrop screening and later runoff screening leads to clear screening effect of erosion. Rainfall prioritizes the transportation of large amount of soils with small particle size over a short period. In the middle stages of rainfall, the soil transportation capacity of runoff is high; however, the transportable soil decreases gradually and soil transport becomes more difficult^21–25^. In addition, it increasingly becomes difficult to transport coarse grains and sand particles on the surface layer^26^, which impedes soil erosion, so that the sheet erosion rate decreases gradually. In the later rainfall stages, soil transport capacity and soil transportable by runoff remain unchanged, and sheet erosion rate remains within a certain range^27^. Owing to the two forces of initial erosion and the uncertainty associated with raindrop erosion, initial erosion has strong volatility. In the later stages, deep runoff reduces the effect of raindrops on erosion volatility^28^. In short, erosion in the red soil in South China first fluctuates and then stabilises with an increase in rainfall duration, as reported previously^29^. Compared to the southern red soil, the loess soil layer of the Loess Plateau is very thick^30^, with mostly fine-grained soil, and the sediment source is sufficient in the later period^31^. Consequently, sheet erosion trends are influenced by soil particle composition and depth of runoff.

Plants can directly affect erosion in three aspects: individual plant characteristics, plant group characteristics, and vegetation layout^32–34^. Under the experimental conditions, the planting layout was consistent under the planting method, and the impact of planting layout on erosion was not considered. Recent studies have shown that plant biomass, plant height, root length, and root density are significantly negatively correlated with erosion^35,36^. Vegetation indirectly influences erosion primarily via reduction of soil erodibility by improving the soil properties^37^. In addition, a certain density of plant groups can effectively shelter rainfall and control and reduce runoff effects. Consequently, the slope erosion rate of Prunella vulgaris was lower. Earthworms reduce erosion by directly improving the characteristics of the individual plants, plant groups, and soil properties. Furthermore, earthworms can increase soil biodiversity, and soil organisms and their derivatives can improve soil properties^38^. In the present study, *P. vulgaris* in combination with earthworms had a lower erosion rate in slopes than the exclusive *P. vulgaris* treatment, indicating that the with earthworms reduced soil erosion.

SOM generally refers to a unique, complex, and relatively stable high-molecular-weight organic compound (humic acid) formed by the action of microorganisms. SOM is an important component of the solid soil and is one of the main sources of plant nutrition. It can promote plant growth and development, improve soil physical properties, promote the activities of microorganisms and soil organisms, and promote the decomposition of nutrients in the soil to improve soil fertility retention and buffering^39^. It is closely related to soil structure, aeration, permeability, adsorption, and buffering^40^. SOM can be used to evaluate the influence of plants, soil, and soil microorganisms on sheet erosion. Therefore, the correlation between soil erosion and SOM could increase in treatments in the order of bare soil slopes, exclusive *P. vulgaris* slopes, and *P. vulgaris* in combination with earthworm slopes.

Slope is a key factor influencing soil erodibility, rain intensity indicates runoff erosivity, and *P. vulgaris* reduces the influence of slope or rain intensity based on the effects of the internal factors associated with sheet erosion^37,41^. The organic matter in the soil can decrease soil erodibility by affecting the infiltration rate and soil roughness^28,35^. Therefore, rain intensity, slope of fallow soil, and SOM in combination can significantly influence sheet erosion characterisation, whereas rain intensity, slope of vegetated slope, and SOM cannot influence sheet erosion characterisation significantly. Similarly, vegetation improves the response to sheet erosion by enhancing soil infiltration and guiding flow^35,41^. Hence, a combination of hydraulic parameters and SOM in bare soil cannot significantly improve sheet erosion characterisation, whereas a combination of SOM and hydraulic parameters in a vegetated slope can significantly improve the characterisation of sheet erosion.

## Conclusion

The high amounts of coarse grains and sand grains in South China soil caused sheet erosion to first decrease and then stabilise with an increase in rainfall duration. SOM improves soil properties and plant growth and its integration in soil erosion equations could improve the characterisation of slope erosion under different treatments. The combination of rain intensity, slope, and SOM in bare soils significantly optimised the sheet erosion equation, and the combination of hydraulic parameters and SOM significantly optimised sheet erosion equations undervegetated slopes.
